# Elevated levels of proinflammatory volatile metabolites in feces of high fat diet fed KK-*A*^*y*^ mice

**DOI:** 10.1038/s41598-020-62541-7

**Published:** 2020-03-30

**Authors:** Misaki Uchikawa, Mai Kato, Akika Nagata, Shunsuke Sanada, Yuto Yoshikawa, Yuta Tsunematsu, Michio Sato, Takuji Suzuki, Tsutomu Hashidume, Kenji Watanabe, Yuko Yoshikawa, Noriyuki Miyoshi

**Affiliations:** 1 School of Food Nutritional Sciences, Shizuoka, Japan; 2 Graduate School of Integrated Pharmaceutical and Nutritional Sciences, Shizuoka, Japan; 3 School of Pharmaceutical Sciences, University of Shizuoka, Shizuoka, Japan; 4 Food Environmental Design Course, Faculty of Education, Art and Science, Yamagata University, Yamagata, Japan; 5 School of Veterinary Medicine, Faculty of Veterinary Science, Nippon Veterinary and Life Science University, Tokyo, Japan

**Keywords:** Biomarkers, Analytical chemistry

## Abstract

When the microfloral composition deteriorates, it triggers low-level chronic inflammation associated with several lifestyle-related diseases including obesity and diabetic mellitus. Fecal volatile organic compounds (VOCs) have been found to differ in gastrointestinal diseases as well as intestinal infection. In this study, to evaluate a potential association between the pathogenesis of lifestyle-related diseases and VOCs in the intestinal tract, fecal VOCs from obese/diabetic KK-*A*^*y*^ mice (KK) or controls (C57BL/6J mice; BL) fed a normal or high fat diet (NFD or HFD) were investigated using headspace sampler-GC-EI-MS. Principal component analysis (PCA) of fecal VOC profiles clearly separated the experimental groups depending on the mouse lineage (KK vs BL) and the diet type (NFD vs HFD). 16 s rRNA sequencing revealed that the PCA distribution of VOCs was in parallel with the microfloral composition. We identified that some volatile metabolites including *n*-alkanals (nonanal and octanal), acetone and phenol were significantly increased in the HFD and/or KK groups. Additionally, these volatile metabolites induced proinflammatory activity in the RAW264 murine macrophage cell line indicating these bioactive metabolites might trigger low-level chronic inflammation. These results suggest that proinflammatory VOCs detected in HFD-fed and/or diabetic model mice might be novel noninvasive diagnosis biomarkers for diabetes.

## Introduction

The intestinal microflora has a marked impact on their host, and the pathophysiology of hosts also influence the intestinal microflora composition, indicating that they communicate with each other^1,2^. Bacterial 16 s rRNA sequencing, a powerful tool to clarify the profile of microflora, has opened new fields and markedly advanced our understanding of the interaction between host and bacteria. A pioneering study reported that obese mice had decreased *Bacteroidetes* with a corresponding increase in *Firmicutes*^3^. This theory was supported by several following studies^4,5^. However, the ratio of *Bacteroidetes* to *Firmicutes* was not completely confirmed in human studies^6–8^, which might be because of differences in genetic and environmental factors such as race, sex, age, and lifestyle.

In addition to 16 s rRNA sequencing, metabolomics analysis is an effective approach to understand the intestinal environment. It was revealed that some bioactive intestinal and microbial metabolites were strongly associated with the etiology of host diseases. For example, deoxycholate generated by the metabolism of intestinal microbiota induced DNA damage and inflammation, which might have a pivotal role in obesity-associated hepatic carcinogenesis^9^. Another example is the metabolites of dietary lipid phosphatidylcholine (lecithin), including trimethylamine *N*-oxide (TMAO) and betaine, whose levels are strongly associated with the pathogenesis of cardiovascular diseases^10,11^. Of note, TMAO and betaine had proinflammatory activities that induced scavenger receptors on macrophages. Similar to compounds such as deoxycholate, TMAO and betaine, bioactive metabolites have advantages as predictive biomarkers. These “bioactive” biomarkers trigger, initiate, and promote pathological events and therefore might be useful for diagnosis in early stage.

Many critical metabolites have been discovered and identified using LC-MS, CE-MS, and NMR. In contrast, another important class of metabolites in the intestinal tract are volatile organic compounds (VOC), which are analyzed by GC-MS. Representative VOCs in the intestinal tract include short-chain fatty acids (SCFAs). SCFAs produced by intestinal fermentation are consumed as an energy source, and function as bioactive metabolites for immune regulation, suppression of food intake, and enhancing intestinal barrier function, which mediate anti-obesity, anti-diabetes, and anti-carcinogenesis effects^12–17^. Therefore, fecal VOCs might be a useful group among intestinal metabolites in the regulation of metabolic homeostasis. Interestingly, fecal VOC analysis was performed to evaluate the pathological features of gastrointestinal diseases^18–20^ including inflammatory bowel disease (IBD)^21–23^, irritable bowel syndrome^21,24^, Crohn’s disease^25^, colorectal cancer (CRC)^26,27^, and necrotizing enterocolitis^28,29^, as well as infectious diseases including paratuberculosis^30–34^, sepsis^35^, giardiasis^36^, and *Clostridium difficile* infection^37^. However, it is difficult to handle intestinal microbial VOCs, because of their unpleasant odor, gaseous form, and low boiling point resulting in their loss when using general sample preparation methods including evaporation or concentration. Previous trials evaluated their odor using an electronic nose device (eNose). Although eNose is an easy and simple way to characterize gaseous samples, it is unavailable for the quali-quantitative profiling of VOCs. Currently, GC-MS is the best method for the identification and quantification of VOCs. Sample preparation for the GC-MS analysis of VOCs requires solid-phase microextraction (SPME), but this method is complicated and unsuitable for multiple analyte samples. Additionally, it requires expensive equipment for the full-automation of the SPME preparation. Therefore, limited numbers of studies on intestinal or fecal VOCs have been reported.

**Figure 1:**
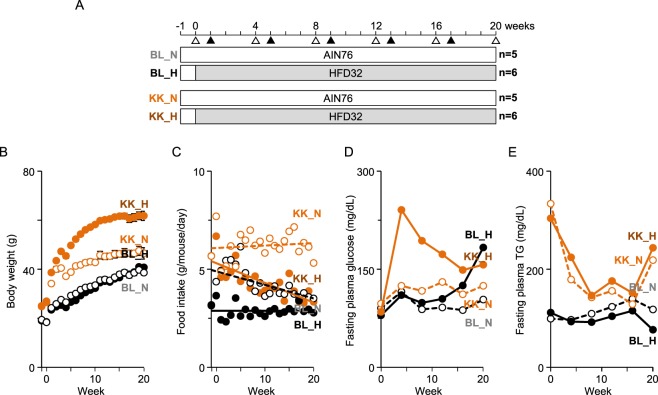
Experimental protocol and anthropometric measurements. (**A**) Experimental protocol used in this study. C57BL/6 mice (BL) and KK-*A*^*y*^ mice (KK) were fed a normal (N) or high fat diet (H), and were grouped as BL_N, BL_H, KK_N, and KK_H, respectively. Blood (Δ) and feces (▲) were collected at the indicated timepoints. Body weight (**B**) and the amount of food intake (**C**) were evaluated every week. Levels of plasma glucose (**D**) and TG (**E**) were determined every 4 weeks.

Chronic inflammation is critical step in the induction of several lifestyle-related diseases. In this study, fecal VOCs from normal fat diet (NFD) or high fat diet (HFD)-fed obese/diabetic KK-*A*^*y*^ (KK) mice or control C57BL/6J (BL) mice (Fig. 1A) were analyzed by headspace sampler-gas chromatography-mass spectrometry (HSS-GC-MS) without the SPME procedure to identify proinflammatory metabolites associated with low-grade inflammation and identify biomarkers reflecting the progression of lifestyle-related disease. Direct analyses of VOCs without SPME condensation and purification is advantageous because sample preparation is simple and there is reduced artificial errors and bias. We detected up to ~80 fecal VOCs in mouse samples and found elevated levels of fecal VOCs such as phenol, acetone, nonanal and octanal were significantly associated with diet type (NFD vs HFD), mouse lineage (BL vs KK), the progression of disease state, and their interaction. These fecal metabolites induced significant proinflammatory activity in mouse RAW264 cells. These results suggest that fecal VOCs might be potential biomarkers for the diagnosis of inflammation-associated lifestyle related diseases.

## Results

### Altered pathophysiological parameters in C57BL/6J and KK-*A*^*y*^ mice

**Figure 2:**
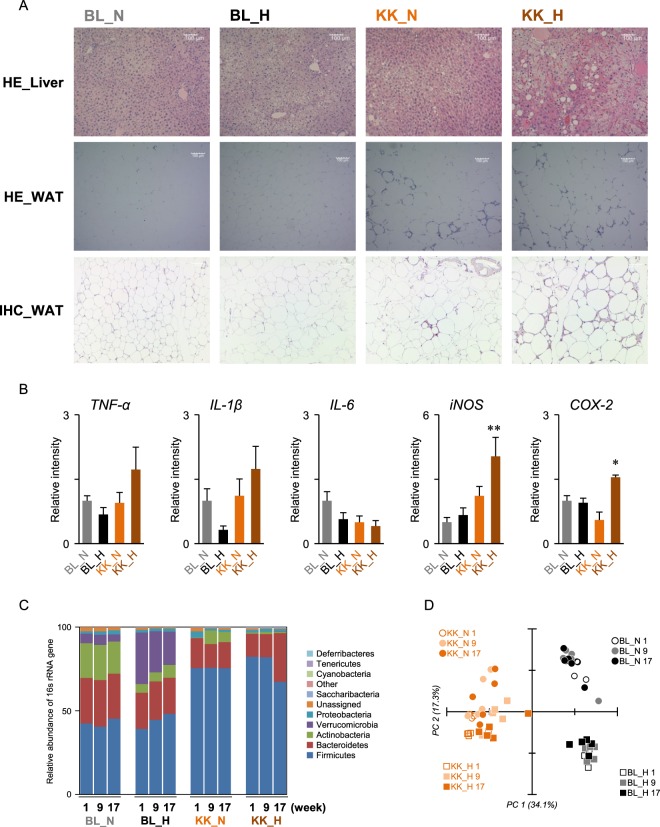
Pathophysiological change in mice liver and feces. (**A**) HE staining and IHC of liver and WAT. Mouse livers and WAT collected at week 20 were prepared and stained with HE and IHC for F4/80. Representative images (scale bar = 100 µm) are shown. (**B**) Inflammatory gene expressions in mouse livers at week 20. RT-qPCR (n = 5–6 in each group) were performed as described in the Materials and Method. *p < 0.05, **p < 0.01 compared with the BL_N group by one-way ANOVA with post hoc test (Bonferroni). (**C**) Microbial composition in mouse feces collected at weeks 1, 9 and 17. 16 s rRNA sequencing was performed. Relative abundance and taxonomic classification at the phylum level were analyzed by QIIME. (**D**) PCA of microbial composition in mouse feces. Data were analyzed by QIIME.

Body weights of KK-*A*^*y*^ mice were significantly higher than C57BL/6J mice for all experimental periods (Fig. 1B). HFD feeding significantly increased body weight in KK-*A*^*y*^ mice, but not in C57BL/6J mice. The amounts of food intake in HFD-fed KK-*A*^*y*^ mice (KK_H) and BL mice (BL_H) were lower than those of the NFD groups (KK_N and BL_N), respectively (Fig. 1C). Elevation of fasting plasma glucose (FPG) at week 4 in KK_H mice was observed, which then declined to ~150 mg/dL at weeks 16–20 (Fig. 1D). The FPG in BL_H mice gradually increased up to ~180 mg/dL at week 20. Although levels of FPG in NFD-fed mice (BL_N and KK_N) were maintained lower than 130 mg/dL, levels in KK_N mice were slightly higher than in BL_N mice. Levels of fasting plasma TG in KK-*A*^*y*^ mice were declined during a feeding period (Fig. 1E). In addition to the ballooning degeneration of hepatocytes in BL_H mice, lipid droplets were detected in KK-*A*^*y*^ mice (Fig. 2A). Additionally, adipocyte hypertrophy and macrophage infiltration were observed in WAT of KK_N and KK_H groups, which would be partially associated with low-level chronic inflammation (Fig. 2A). Although the hepatic gene expressions of TNF-α, IL-1β, and IL-6 at week 20 were not significantly altered among the groups, iNOS and COX-2 gene expressions were significantly increased in KK-*A*^*y*^ and KK_H mice, respectively (Fig. 2B). 16 s rRNA sequencing revealed reduced levels of *Bacteroidetes* whereas an increase in *Firmicutes* was observed in KK-*A*^*y*^ mice (Fig. 2C). Principal component analysis (PCA) of the 16 s rRNA sequencing showed that the mouse lineage was clearly separated depending on the first principal component (PC1) (Fig. 2D). An association of the second principal component (PC2) with the type of diet (NFD or HFD) was observed, especially in C57BL/6J mice compared with KK-*A*^*y*^ mice. No clear correlation with time-axis during weeks 1–17 was observed on PC1 and PC2. Therefore, in the current experimental conditions, diet-induced microbial compositional change was probably constructed within the first week (weeks 0–1) rather than during the following experimental period.

### Fecal VOC profile in NFD- or HFD-fed C57BL/6J or KK-*A*^*y*^ mice

**Figure 3:**
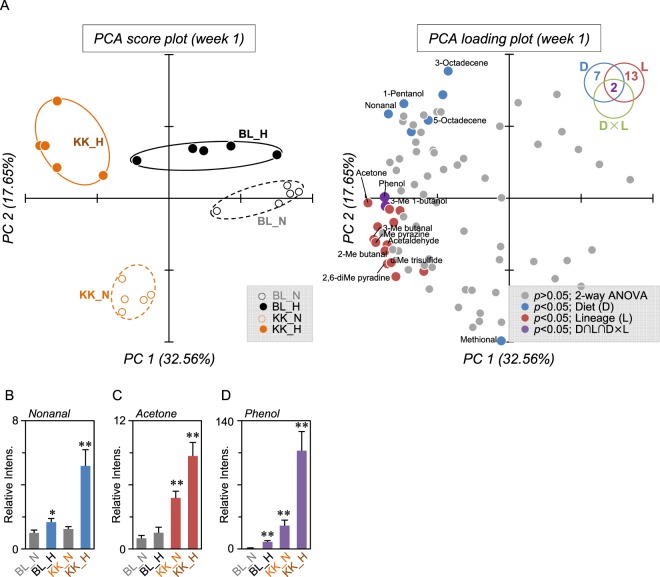
Fecal VOC profile in NFD- or HFD-fed C57BL/6J or KK-*A*^*y*^ mice. (**A**) PCA score plot and loading plot of fecal VOC analysis at week 1. The peak area values of the integrated 82 peaks obtained by GC-MS analyses were used as variables in PCA. The diagram in the upper right summarizes the quantity of significance (*p* < 0.05) by two-way ANOVA with multiple testing correction using the Bonferroni family-wise error rate. Compounds determined as significantly different by diet (D) and mouse lineage (L) are shown as blue and red dots, respectively. Two compounds, phenol and 3-methyl 1-butanol (purple dots), were determined as significant in D × L (interaction between L and D by two-way ANOVA) and in L and D (D ∩ L ∩ D × L). Other compound peaks not determined as significant are shown as gray dots. Ellipses drawn on PCA score plot do not reflect any statistical significance. Relative fecal levels of nonanal (**B**), acetone (**C**), and phenol (**D**) in C57BL/6 and KK-*A*^*y*^ mice fed a normal or high fat diet are summarized. Data are the means ± SEM (n = 5). Significant difference; *p < 0.05, **p < 0.01 compared with BL_N by one-way ANOVA with post hoc test (Bonferroni).

To identify volatile biomarkers that reflected the pathophysiological alterations in NFD or HFD-fed C57BL/6J or KK-*A*^*y*^ mice, feces were analyzed by HSS-GC-MS (Supplemental Fig. 1). Raw data were subjected to AMDIS, then the extracted peaks were integrated and filtered by frequency (80% presence in at least one group), resulting in the integration of MS ion peaks to 82, 57, 49, 49, and 36 entities at weeks 1, 5, 9, 13 and 17, respectively (Supplemental Table 1–5). Each integrated data set was applied to PCA and two-way ANOVA. Figure 3 shows the results at week 1. PCA (score plot) clearly separated the groups depending on the mouse lineage (PC1 on the x-axis) and the influence of diet type (PC2 on the y-axis). Seven and 13 entities were determined as having a significant difference in diet (D) and mouse lineage (L) by two-way ANOVA (Fig. 3A and Table 1). The strongest contributor on the y-axis (showing the biggest value of PC2) was methional, which had lower levels after HFD feeding (Table 1). However, among the nine entities (*p* < 0.05 in D), nonanal was placed furthest away from the origin in the loading plot, and was increased in the HFD groups (Fig. 3B). Although a small amount of nonanal detected in HSS-GC-MS might have been formed by heating during sample preparation, LC-MS analysis confirmed a higher level of fecal nonanal, indicating fecal fat heating in HSS (100 °C for 60 min) has little effect on the degradative production of nonanal (Supplemental Fig. 2). Additionally, LC-MS analysis confirmed that octanal and heptanal were also increased in HFD groups (Supplemental Fig. 2). Acetone was the strongest contributor on the x-axis, having the longest distance from the origin in the loading plot within 15 entities (*p* < 0.05 in L), indicating it was increased in KK-*A*^*y*^ mice (Fig. 3C). Additionally, levels of two compounds, phenol and 3-methyl butanol, were significant in D and L, and also in the interaction between D and L. Levels of phenol were significantly increased by HFD, mouse lineage (KK group), and these interactions (Fig. 3D). Notably, levels of phenol in KK_H groups were 107-fold higher than that of BL_N.

**Table 1:** VOCs determined as significantly different (*p* < 0.05) in mouse feces at week 1.

RT (min)	Base peak	Name	PC 1	PC 2	*p* (two-way ANOVA)^*^		
			(32.56%)	(17.65%)	Diet	Lineage	D × L
1.66	29	Acetaldehyde	−3.57	−1.30		7.8E-03	
2.14	43	Acetone	−4.13	−0.13		2.2E-03	
2.16	41	*Unidentified*	−3.50	−0.31		4.5E-04	
3.04	41	2-Methyl-butanal	−3.63	−1.46		3.6E-04	
3.06	44	3-Methyl-butanal	−3.96	−1.15		4.1E-03	
3.46	126	5-Octadecene	−3.31	−2.18		3.9E-03	
3.91	43	*Unidentified*	−3.38	−0.67		2.5E-02	
10.08	55	3-Methyl-1-butanol	−3.61	−0.22	4.9E-03	4.0E-04	2.3E-02
10.31	55	1-Pentanol	−3.08	2.63	1.7E-03		
10.52	94	Methyl pyrazine	−3.90	−1.23		4.3E-03	
12.07	108	2,6-Dimethyl pyradine	−3.56	−1.82		4.8E-06	
12.61	108	*Unidentified*	−3.19	−0.34		3.7E-02	
13.17	126	Dimethyl trisulfide	−3.45	−1.79		1.9E-05	
13.66	57	Nonanal	−3.53	2.34	1.7E-03		
15.25	48	Methional	−0.23	−3.96	1.1E-03		
16.17	120	*Unidentified*	−2.50	−2.03		4.1E-02	
16.39	57	*Unidentified*	−2.86	1.85	3.3E-02		
19.49	61	*Unidentified*	−3.84	−0.79		7.1E-04	
19.65	82	*Unidentified*	−1.94	2.88	1.0E-06		
20.12	83	3-Octadecene	−1.79	3.54	1.2E-21		
20.91	83	5-Octadecene	−2.37	2.17	1.9E-04		
22.05	94	Phenol	−3.64	0.03	2.3E-14	1.3E-15	1.7E-12

*Statistical analysis was performed by two-way ANOVA with multiple testing correction using the Bonferroni family-wise error rate. Significant differences (*p *< 0.05) in diet (D), mouse lineage (L), or the interaction between D and L (D × L) are shown.

**Figure 4:**
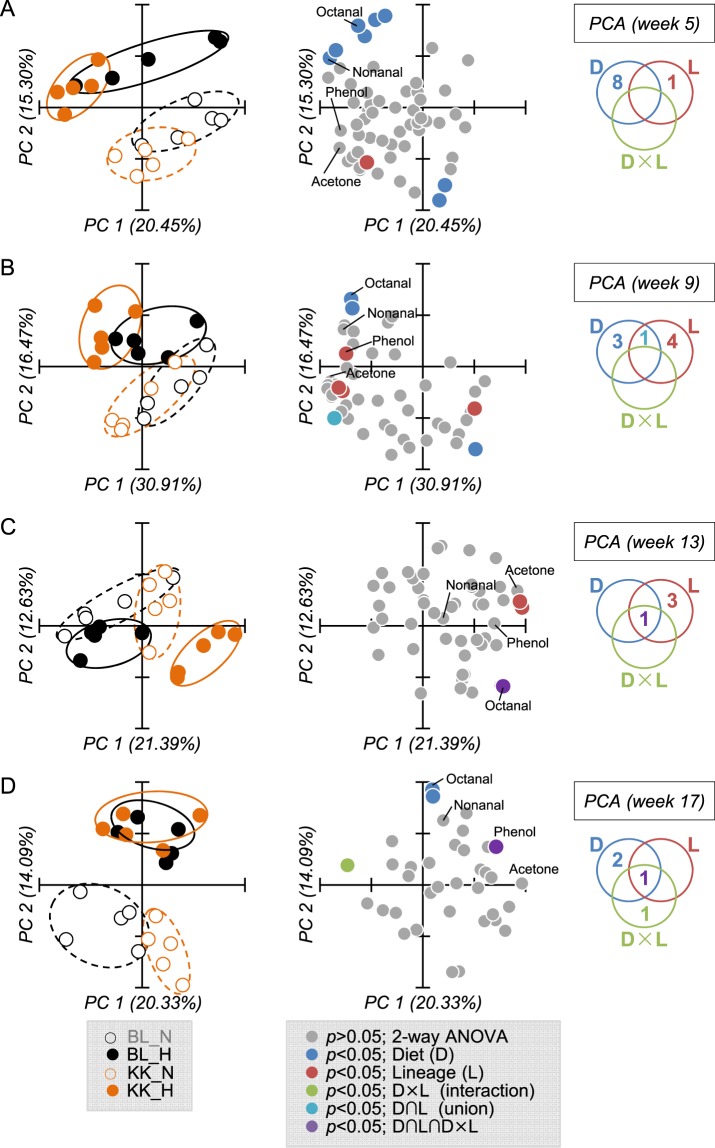
Fecal VOC profile at weeks 5 (**A**), 9 (**B**), 13 (**C**), and 17 (**D**). Feces collected from C57BL/6 and KK-*A*^*y*^ mice fed with a normal or high fat diet were subjected to HSS-GC-MS, then the integrated peak area was used as a variable in PCA. Results show the score plot (left), loading plot (center), and a diagram (right) summarizing the significance (*p* < 0.05) by two-way ANOVA with multiple testing correction using the Bonferroni family-wise error rate. Ellipses drawn on PCA score plot do not reflect any statistical significance.

Further analyses were performed to obtain fecal VOC profiles for weeks 5, 9, 13, and 17 (Fig. 4). Results of PCA and two-way ANOVA are listed in Supplemental Tables 2–5. PCA score plots constructed with PC1 and PC2 showed that better separation was observed by diet type than mouse lineage. We found 4–9 entities (fecal VOCs) at weeks 5, 9, 13, and 17 were statistically extracted as significant (*p* < 0.05). Fecal VOC profiles showed that phenol was statistically significant (*p* < 0.05) in D, L, and D × L at weeks 1 and 17, and that lipid aldehydes including nonanal or octanal were statistically significant (*p* < 0.05) in D at weeks 1–17 (Fig. 4 and Supplemental Fig. 3). PCA loading plots indicated that phenol, acetone, and lipid aldehydes including nonanal and octanal might be candidate biomarkers to determine pathophysiological changes in C57BL/6J and KK-*A*^*y*^ mice.

### Proinflammatory activities of fecal VOCs in RAW264 cells

**Figure 5:**
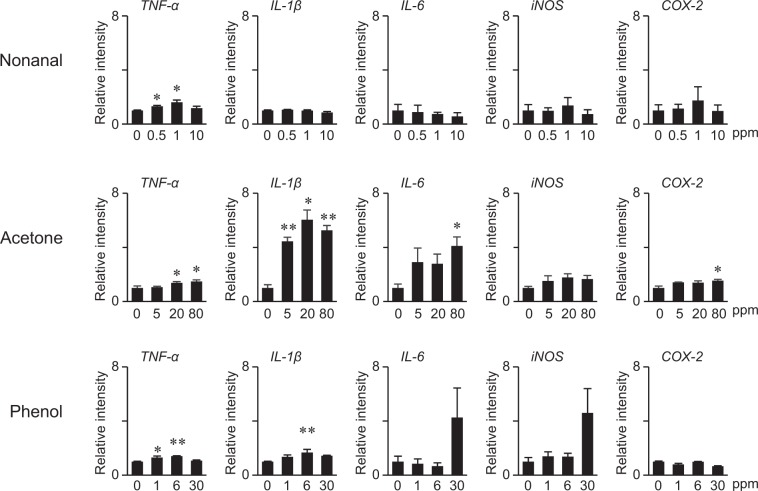
RT-qPCR analysis for proinflammatory activities of VOCs in RAW264 cells. RAW264 cells were treated with nonanal, acetone, or phenol at the indicated concentrations for 6 hours. Relative gene expression levels were analyzed by qRT-PCR. Values are the mean ± SEM (n = 3). Statistical analyses were performed by one-way ANOVA followed by post hoc test (Bonferroni). Statistical significance was set as *p < 0.05, **p < 0.01 compared with controls (0 ppm).

Levels of nonanal, acetone, and phenol in feces collected from HFD-fed KK-*A*^*y*^ mice at week 1 were estimated as 3.8, 20, and 5.9 ppm (≒ mg/kg) by HSS-GC-MS analysis. Additionally, LC-MS analysis revealed that levels of nonanal in plasma collected from HFD-fed KK-*A*^*y*^ mice at week 20 were 2.68 ± 0.09 µM. Therefore, mouse RAW264 cells were exposed to nonanal, acetone, or phenol at the indicated concentration range to evaluate the proinflammatory activities of fecal VOCs. As shown in Fig. 5, TNF-α was slightly but significantly upregulated by nonanal, acetone, and phenol at concentrations achievable *in vivo*. IL-1β was significantly induced by acetone and phenol but not nonanal at appropriate concentrations. Although IL-6, iNOS and COX-2 were dose-dependently induced by acetone, significance was only observed for IL-6 and COX-2 when exposed to high doses. High dose phenol induced IL-6 and iNOS, but this was not statistically significant, when compared with control (0 µM).

## Discussion

In this study, we performed HSS-GC-MS analyses of mice feces to determine fecal VOC profiles. We identified VOCs including *n*-alkanals (nonanal and octanal), acetone and phenol, which were essential metabolites that defined the experimental groups divided by diet type (NFD or HFD) and diabetic state. *n*-alkanals (nonanal and octanal) represented HFD feeding, acetone a major metabolite in diabetic animals, and phenol was synergistically increased by HFD and diabetes. Additionally, these VOCs induced proinflammatory activities and therefore are bioactive metabolites. HSS-GC-MS analysis of fecal VOCs combined with bioassay (RT-qPCR) will have an important role in metabolomics to discover noninvasive diagnosis biomarkers.

Nonanal (C_9_H_18_O), and octanal (C_8_H_16_O) are lipid aldehydes classified as *n*-alkanals, which are formed *in vivo* via host or bacterial metabolism or the auto oxidation of unsaturated fatty acids. We previously developed a comprehensive quantitation method for lipid aldehydes using LC-ESI-MS/MS after derivatization with dansyl hydrazine and reported that levels of nonanal, octanal and heptanal (C_7_H_14_O) in C57BL/6J mouse plasma were 0.65, and 0.13, and 0.06 µM, respectively^38^. These lipid aldehydes, therefore, seem to be ubiquitously distributed in the living body. In this study, these lipid aldehydes were also quantified by LC-MS. Levels of nonanal in plasma collected at week 20 (sacrifice) were 2.22 and 2.37 µM in BL_N and BL_H groups, and 2.41 ad 2.68 µM in KK_N and KK_H groups, respectively. The levels of octanal were 0.09 and 0.10 µM in BL_N and BL_H groups, and 0.10 and 0.11 µM in KK_N and KK_H groups, respectively. Therefore, these lipid aldehydes were increased in the feces and plasma by HFD feeding. Although nonanal is an abundant lipid aldehyde in feces and plasma, octanal might be a precision biomarker to distinguish feeding with a fatty diet. Nonanal within a clinically achievable concentration range significantly increased TNF-α in RAW264 cells (Fig. 5). Other studies also support the proinflammatory effects of nonanal and octanal^39^. These results suggest that nonanal and octanal metabolites might induce low-level chronic inflammation via the induction of inflammatory cytokines.

Acetone is the simplest and smallest ketone with a molecular formula (CH_3_)_2_CO, boiling point of 56 °C, oral LD50 values in adult rats in the range 5800–7138 mg/kg, and is not considered carcinogenic. Acetone is formed in the living cells of animals, plants, and microorganisms, and therefore is ubiquitously detectable in biological samples. For example, the mean concentration was 800 ng/L in the expired breath of healthy children^40^, and was 3,100 ppb in the blood of 600 non-occupationally exposed US people^41^. One of the major metabolisms to produce acetone in humans is the ketone body formation pathway. Ketone bodies consisting of acetone, acetoacetic acid, and β-hydroxybutyric acid, are formed in the liver under conditions of unusable glucose (carbohydrate) or hard to use conditions (fasting, carbohydrate restrictive diet, untreated diabetes) to supply the fuel to outer organs such as the brain. Although acetoacetic acid and β-hydroxybutyric acid are enzymatically convertible with each other, acetone is irreversibly converted from acetoacetic acid, and is considered a metabolic end product. It was reported that higher levels of acetone were detected in the expired breath of diabetic patients^42,43^. Acetone is membrane permeable and therefore can diffuse throughout the entire body including the breath and feces. Bacteria also produce acetone via metabolism. Recent reports suggested that increased levels of acetone in the feces correlated with specific bacterial species in inflammatory bowel disease (IBD) and cystic fibrosis^44^. We observed that acetone strongly induced IL-1β, IL-6, and COX-2 (Fig. 5). Therefore, acetone derived from microbial-host co-metabolism might systemically trigger low-level chronic inflammation.

Phenol, monohydroxy benzene, is an organic compound with the molecular formula C_6_H_5_OH and boiling point of 181.7 °C. Phenol is not carcinogenic but induces toxicity: LD50 values in mice are 112 mg/kg (i.v.) and 270 mg/kg (oral). In the current study, GC-MS analyses of feces at week 1 revealed that phenol was significantly increased by HFD in the KK group and these interactions (Fig. 3D). The levels of fecal phenol in BL_HFD and KK_NFD groups gradually increased up to 17 weeks (Fig. S3). Since the fecal level has wide dynamic range as shown in Fig. 3D, phenol could be a promising biomarker to monitor the clinical condition of diabetes. It was reported that some intestinal bacteria such as *Escherichia coli*, *Proteus* species, *Bacteroides fragilis*, *Clostridium* species, and *Staphylococcus* species, metabolize tyrosine to phenol^45,46^. 16 s rRNA sequence analysis indicated that among these bacteria, *Staphylococcus* had a weak correlation with the fecal level of phenol. In addition to bacterial populations, the dietary protein ratio in HFD32 (30%) compared with AIN76 (20%) might contribute to the increased production of phenol. Some phenol produced by intestinal bacteria is excreted in the feces and was detected by HSS-GC-MS analyses. The rest of the phenol was absorbed by the colon, conjugated with sulfate, and circulated through the bloodstream. We found that plasma levels of phenol were synergistically increased by HFD and diabetes (data not shown). It was reported that phenol produced by intestinal bacteria cause skin problems by disrupting keratinization^47^, and therefore might spread around the body. Phenol had proinflammatory effects on murine macrophage RAW264 cells (Fig. 5). Taken together, phenol might systemically trigger low-level chronic inflammation.

Fecal VOCs might be potential biomarkers for clinical diagnosis. Many VOCs such as acetone, phenol and *n*-alkanals, are membrane permeable because of their chemical properties such as small size and light weight. Similar to acetone, some VOCs detected in the feces were probably generated by the host rather than intestinal bacteria. These metabolites penetrate throughout the body and are detectable in the feces and blood. Fecal VOCs analysis is a noninvasive method and might aid the development of diagnosis biomarkers for several diseases. Small VOCs with a low boiling point similar to acetone might be detectable in expired breath, which is a safe and easy-to-use approach. In addition, VOC analyses of the cecum or intestinal contents may provide valuable information because it is a more relevant analyte for understanding the intestinal environment.

In this study we used HSS-GC-EI-MS, which has two advantages for metabolomics. First, it is easy to identify compounds because a well-established database for EI-MS spectra is available. Second, sample preparation is simple, requiring feces in a vial sealed with a barotolerance cap and set on HSS, which prevents unpleasant odors. Technical difficulties still limit the widespread use of VOC analysis in clinical settings, but this approach has already been applied successfully for the diagnosis of CRC^48^. Fecal VOC analysis might be a promising approach to detect gastrointestinal and other diseases. Basic analytical tools used for the detection of volatiles include GC, MS, GC-MS, chemiluminescence, optical absorption spectroscopy systems, electronic noses, and different types of gaseous sensors. The identification of noninvasive biomarkers by fecal VOCs analyses is comparatively recent and a standardized method has not been developed.

In this study, we performed VOC analysis of mice feces and obtained a VOC profile dependent on diet and clinical conditions, which allowed us to propose candidate biomarkers. These VOCs including nonanal, acetone, and phenol induced proinflammatory effects. Therefore, they were bioactive metabolites and might be noninvasive diagnosis biomarkers in early stage. Although nonanal and acetone are already known to be formed under the conditions of oxidative stress and diabetes, the formation mechanism of phenol is not fully understood. Because microbiota are important for maintaining a healthy state as well as promoting disease, an understanding of the mechanisms of fecal volatile biomarker formation, such as phenol, might be a novel and potential target for preventive and therapeutic strategies. Further analyses are required to determine the levels of these metabolites and other gaseous candidates in other diabetes animal models and humans using a highly accurate quantitative analytical method for clinical applications.

## Materials and Methods

### Animal experiments

All animal experiments were approved by the animal ethics committee of University of Shizuoka (approval number 135005), and performed according to guidelines for the care and use of laboratory animals at the University of Shizuoka. Five-week-old male KK-*A*^*y*^ and C57BL/6J mice were purchased from CLEA Japan Inc. (Tokyo, Japan). All animals were housed individually in plastic cages and had free access to drinking water under controlled conditions of humidity (55 ± 5%), light (12/12-h light/dark cycle) and temperature (23 ± 1 °C). After a 1-week adaptation period with a basal diet (AIN-76, Oriental Yeast, Co., Ltd., Tokyo, Japan) given *ad libitum*, mice were randomized by body weight into normal fat diet (NFD, n = 5) and high fat diet (HFD) groups, and then a NFD (AIN-76) was given to C57BL_NFD (n = 5) and KK-*A*^*y*^_NFD (n = 5) mice or a HFD (HFD32, CLEA Japan Inc.) was given to C57BL_HFD (n = 6) and KK-*A*^*y*^_HFD (n = 6) mice until the end of the experiment (Fig. 1A, Table S1). Blood samples were collected from the tail vein every 4 weeks using heparinized capillary tubes, and the levels of triglyceride were determined using the Triglyceride E test (Wako Pure Chemical, Japan). Levels of blood glucose were monitored using ACCU-Chek Aviva (Roche, Indianapolis, IN). One week after the blood collection, mice were placed into a metabolic cage for 24 hours and their feces were collected. Aliquots (100 mg) of feces were immediately subjected to HSS-GC-MS. Liver and WAT collected at week 20 were examined by hematoxylin-eosin (HE) staining, and immunohistochemistry.

### Cell culture

RAW264 cells (RIKEN Cell Bank, Ibaraki, Japan) were maintained in 10% FBS/DMEM supplemented with 50 U/ml penicillin and 50 µg/ml streptomycin and grown in an atmosphere of 95% air and 5% CO_2_ at 37 °C.

### Real-time RT-PCR

Total RNA extracted using TRIzol reagent (Invitrogen) was converted into cDNA using PrimeScript RT Master Mix (TaKaRa). To quantitatively estimate the level of each gene, quantitative PCR was performed using gene-specific primers, cDNA and SYBR Premix (TaKaRa). The sequences of the PCR primer pairs are as follows: *18 s rRNA*; 5′-CTT CTC CAT GTC GTC CCA GT-3′ and 5′-ACG CTG AGC CAG TCA GTG TA-3′, *TNF-α*; 5′-GAT TAT GGC TCA GGG TCC AA-3′ and 5′-CCC AGC ATC TTG TGT TTC TG-3′, *IL-1β*; 5′-TCT TCC TAA AAG TAT GGG CTG GA-3′ and 5′-AAA AGG GAG CTC CTT AAC ATG C-3′, *IL-6*; 5′-CGC TAT GAA GTT CCT CTC TGC-3′ and 5′-TTG GGA GTG GTA TCC TCT GTG-3′, *iNOS*; 5′-GGT ATG CTG TGT TTG GCC TTG-3′ and 5′-TTC GTC CCC TTC TCC TGT TG-3′, and *COX-2*; 5′-GGA GGC GAA GTG GGT TTT AAG-3′ and 5′-TTG ATG GTG GCT GTT TTG GTA G-3′.

### NGS 16 s sequencing

Microbial DNA in feces was extracted and the V3-V4 variable region of 16 s rRNA was amplified using a specific primer (5′-TCG TCG GCA GCG TCA GAT GTG TAT AAG AGA CAG CCT ACG GGN GGC WGC AG-3′ and 5′-GTC TCG TGG GCT CGG AGA TGT GTA TAA GAG ACA GGA CTA CHV GGG TAT CTA ATC C-3′). Quantified amplicons were tagged with a barcode and then sequenced using MiSeq (Illumina, San Diego, CA, USA). Data were analyzed by Nephele (National Institutes of Health, Bethesda, MD, USA) and QIIME^49^.

### HSS-EI-GC-MS

Fecal VOCs were analyzed by HSS-GC-MS consisting of Agilent 7697 headspace sampler, Agilent 7890 gas chromatography, and an Agilent 5975 mass spectrometer. Fecal samples (100 mg) in a barotolerance vial were heated in HSS at 100 °C for 60 min, then injected (50 ml/min for 1 min) with a split ratio (5:1) to GC through the inlet maintained at 250 °C. VOCs in samples were separated by a DB-WAX column (30 m, 0.25 mm, 0.25 µm), at an initial temperature of 35 °C for 1 min, which was increased by 5 °C/min to 120 °C, and then maintained at 250 °C for 10 min. Helium was used as the carrier gas at 1.1 mi/min constant flow. Ion source (EI) and quadrupole were maintained at 230 °C and 150 °C, respectively. VOCs detected between *m/z* 14–500 were monitored. MS peak data from HSS-GC-MS analyses were subjected to AMDIS software for peak detection and integration, and Mass Profiler Professional software (Agilent Technology) for data processing and principal component analysis. The levels of phenol, acetone, and nonanal in feces detected by GC-MS were estimated by external standard methods. For the external standard curves, references standards of nonanal, acetone and phenol sampled using glass capillary in barotolerance vials were injected to HSS-GC-MS.

### LC-ESI-MS/MS

LC-MS analyses were performed to quantify *n*-alkanals (nonanal, octanal and heptanal) in feces and serum as described previously^38^. Briefly, 30 mg feces or 20 µl plasma were extracted using CHCl_3_/MeOH and then derivatized with dansyl hydrazine (DH). LC-MS/MS analyses were performed on an Agilent 1290 series HPLC coupled with a G6410B triple quadrupole tandem mass spectrometer. HPLC separation was performed with a TSKgel super-Octyl column (2.3 µm, 100 × 2.0 mm, TOSOH) at 40 °C. DH derivatives were detected by monitoring their transition [M + H]^+^ -> 236.1 in MRM mode.

### Statistical analysis

All data are presented as the mean ± SEM. All statistical analyses were performed with EZR (Saitama Medical Center, Jichi Medical University), a graphical user interface for R (The R Foundation for Statistical Computing). Statistical analyses of data were performed by one-way ANOVA followed by Bonferroni’s post hoc test and two-way ANOVA with multiple testing correction using the Bonferroni family-wise error rate. Differences were considered significant when *p* < 0.05.

## Supplementary information

Supplementary Table 6

Supplementary Figure S1

Supplementary Figures S2

Supplementary Figure S3

Supplementary Table 1

Supplementary Table 2

Supplementary Table 3

Supplementary Table 4

Supplementary Table 5
