# Supplementary material for: Elevated levels of proinflammatory volatile metabolites in feces of high fat diet fed KK-Ay mice
Source: Sci Rep. 2020 Mar 30;10:5681. doi: 10.1038/s41598-020-62541-7 (PMC7105489; doi:10.1038/s41598-020-62541-7)
Supplement: Supplementary file 1 — Supplementary Table 6 [file 41598_2020_62541_MOESM1_ESM.pdf]

Supplemental Table 6. Dietary composition of AIN76 and HFD32 used in this study

AIN76 Ingredient	(%)	HFD32 Ingredient	(%)
Milk Casein	20	Milk Casein	24.5
DL-methionine	0.3	Egg white	5
		L-cystine	0.43
Corn oil	5	Beef tallow powder	15.88
		High oleic acid safflower oil	20
Corn starch	15	Cellurose	5.5
Sucrose	50	Maltdextrin	8.25
Cellurose powder	5	Lactose	6.928
		Sucrose	6.75
AIN-93 vitamin mix	1	AIN-93 vitamin mix	1.4
AIN-93G mineral mix	3.5	AIN-93 mineral mix	5
Choline bitartrate	0.2	Choline bitartrate	0.36
		t-Butylhydroquinone	0.002
	100		100
