# Supplementary material for: Elevated levels of proinflammatory volatile metabolites in feces of high fat diet fed KK-Ay mice
Source: Sci Rep. 2020 Mar 30;10:5681. doi: 10.1038/s41598-020-62541-7 (PMC7105489; doi:10.1038/s41598-020-62541-7)
Supplement: Supplementary file 2 — Supplementary Figure S1 [file 41598_2020_62541_MOESM2_ESM.pdf]

Figure S1. Uchikawa *et al.*

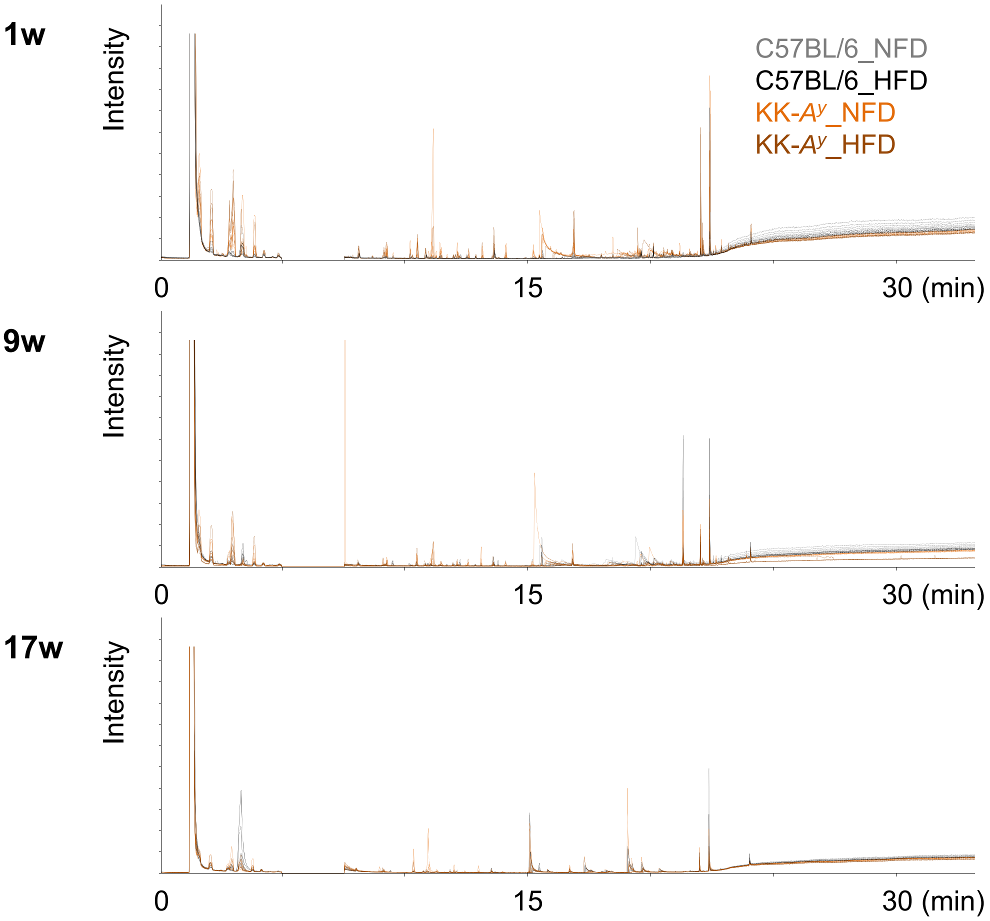

Figure S1. Typical chromatograms of HSS-GC-MS analyses for mouse feces collected from C57BL/6_NFD (gray, n=5), C57BL/6_HFD (black, n=5), KK-A^y_NFD (orange, n=5), and KK-A^y_HFD (brown, n=5) groups at weeks 1, 9, and 17.
