# Supplementary material for: Elevated levels of proinflammatory volatile metabolites in feces of high fat diet fed KK-Ay mice
Source: Sci Rep. 2020 Mar 30;10:5681. doi: 10.1038/s41598-020-62541-7 (PMC7105489; doi:10.1038/s41598-020-62541-7)
Supplement: Supplementary file 3 — Supplementary Figures S2 [file 41598_2020_62541_MOESM3_ESM.pdf]

Figure S2. Uchikawa *et al.*

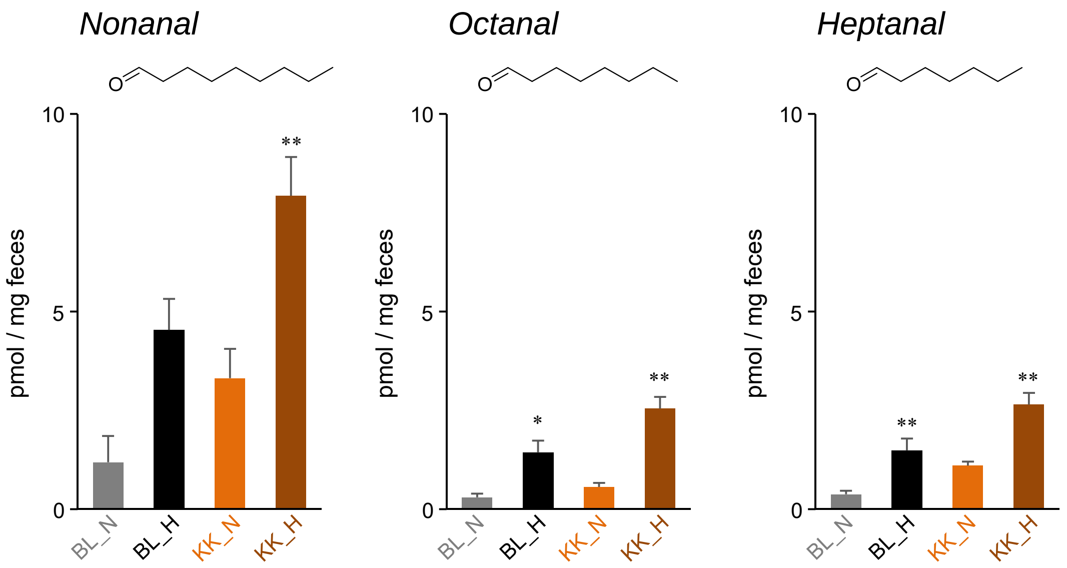

Figure S2. LC-MS analysis of fecal lipid aldehyde. To exclude the possibility that nonanal was artificially generated from fecal fat by heating in HSS at 100° C for 60 min, feces collected at week 1 from BL_N (gray, n=5), BL_H (black, n=5), KK_N (orange, n=5), and KK_H (brown, n=5) groups were prepared by $CHCl_3$/MeOH extraction without heating. Samples were derived with dansyl hydrazine, then subjected to LC-MS[39]. Data are the means ± SEM (n=5). Significant difference; *$p < 0.05$, **$p < 0.01$ compared with BL_N (gray bar) at each week by one-way ANOVA with post hoc test (Bonferroni). High levels of nonanal, octanal and heptanal were detected by LC-MS analyses, indicating that the high level of nonanal detected by HSS-GC-MS was not a heat degradation product of fecal fat.
