# Supplementary material for: Elevated levels of proinflammatory volatile metabolites in feces of high fat diet fed KK-Ay mice
Source: Sci Rep. 2020 Mar 30;10:5681. doi: 10.1038/s41598-020-62541-7 (PMC7105489; doi:10.1038/s41598-020-62541-7)
Supplement: Supplementary file 4 — Supplementary Figure S3 [file 41598_2020_62541_MOESM4_ESM.pdf]

Figure S3. Uchikawa *et al.*

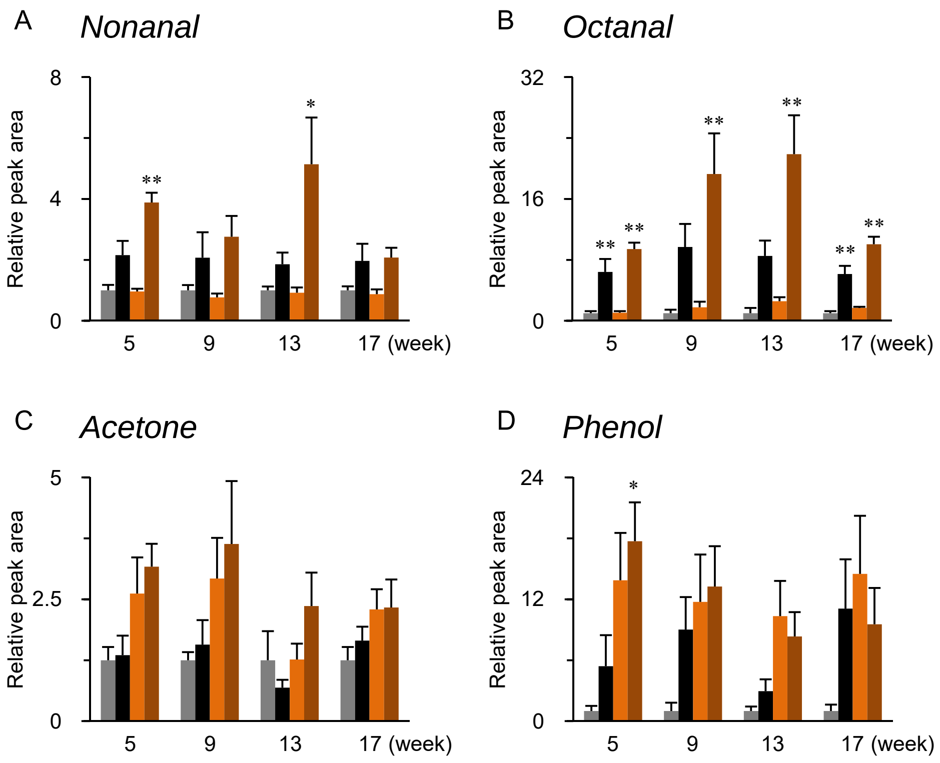

Figure S3. Fecal levels of nonanal (A), octanal (B), acetone (C), and phenol (D). VOCs in feces collected from BL_N (gray), BL_H (black), KK_N (orange), and KK_H (brown) groups at the indicated timepoints were determined by HSS-GC-MS. Relative peak areas were normalized to a value of 1 for BL_N at week 5. Data are the means ± SEM (n=5). Significant difference; **p* < 0.05, ***p* < 0.01 compared with BL_N (gray bar) at each week by one-way ANOVA with post hoc test (Bonferroni).
