# Supplementary material for: Elevated levels of proinflammatory volatile metabolites in feces of high fat diet fed KK-Ay mice
Source: Sci Rep. 2020 Mar 30;10:5681. doi: 10.1038/s41598-020-62541-7 (PMC7105489; doi:10.1038/s41598-020-62541-7)
Supplement: Supplementary file 5 — Supplementary Table 1 [file 41598_2020_62541_MOESM5_ESM.pdf]

Supplemental Table 1. List of VOCs analyzed by PCA at week 1.

RT (min)	Base peak	Name	PC 1 (32.56%)	PC 2 (17.65%)	*p* (two-way ANOVA) Diet	Lineage	DL
1.43	28		0.84	1.50			
1.44	252		2.02	1.36			
1.46	32		1.54	1.85			
1.46	45		3.53	0.52			
1.49	31		-1.92	0.80			
1.50	58		0.72	-1.76			
1.52	17		-1.52	0.92			
1.61	47		-2.23	1.33			
1.66	29	Acetaldehyde	-3.57	-1.30		7.8E-03	
1.73	236		2.63	0.94			
2.14	43	Acetone	-4.13	-0.13		2.2E-03	
2.16	41		-3.50	-0.31		4.5E-04	
2.17	43	2-Methyl-propanal	2.10	-2.45			
2.58	82		-2.00	2.40			
2.87	43	2-Butanone	-3.30	0.77			
2.88	43		-2.39	0.62			
2.89	31	3-Methyl-1-butanol	-0.71	0.56			
2.94	31	1-Pentanol	-0.23	0.01			
3.04	41	2-Methyl-butanal	-3.63	-1.46		3.6E-04	
3.06	44	3-Methyl-butanal	-3.96	-1.15		4.1E-03	
3.37	31		0.26	2.66			
3.38	31	Ethanol	1.39	2.89			
3.45	97	3-Octadecene	-3.40	-1.43			
3.46	126	5-Octadecene	-3.31	-2.18		3.9E-03	
3.91	43		-3.38	-0.67		2.5E-02	
3.91	43	2-Pentanone	-2.73	1.56			
4.29	41	Acetonitrile	-0.28	2.00			
4.88	75	Methional	-1.33	-0.92			
4.99	28		-2.18	1.27			
7.57	18		-0.25	-1.34			
8.15	70	Heptanal	-2.77	0.38			
8.54	79	Pyridine	-2.58	1.83			
8.70	84	3-Methyl-2-butenal	-2.35	-1.69			
9.22	81	2-Pentyl-furan	-1.33	1.10			
10.08	55	3-Methyl-1-butanol	-3.61	-0.22	4.9E-03	4.0E-04	2.3E-02
10.24	56	3-Methyl-3-buten-1-ol	-1.09	-2.56			
10.31	55	1-Pentanol	-3.08	2.63	1.7E-03		
10.52	94	Methyl pyrazine	-3.90	-1.23		4.3E-03	
11.13	45	Acetoin	-2.34	-2.53			
11.48	43	1-Hydroxy-2-propanone	-3.23	-1.47			
12.07	108	2,6-Dimethyl pyradine	-3.56	-1.82		4.8E-06	
12.54	341		1.69	-1.73			
12.61	108		-3.19	-0.34		3.7E-02	
12.90	56	1-Hexanol	-3.21	1.09			
13.17	126	Dimethyl trisulfide	-3.45	-1.79		1.9E-05	
13.57	58	2-Nonanone	-3.10	2.12			
13.66	57	Nonanal	-3.53	2.34	1.7E-03		
14.13	122	Trimethyl pyrazine	-2.25	-1.92			
15.25	48	Methional	-0.23	-3.96	1.1E-03		
15.48	96	Furfural	-0.85	-3.20			
15.49	70	Heptyl formate	-2.50	2.31			

15.78	60	Acetic acid	-1.07	-3.59			
16.17	120		-2.50	-2.03		4.1E-02	
16.39	57		-2.86	1.85	3.3E-02		
16.84	281		1.34	-2.24			
16.89	106	Benzaldehyde	-3.69	-0.97			
17.51	55	1-Dodecanol	-3.11	2.14			
18.01	56	1-Octanol	-3.08	2.28			
19.49	61		-3.84	-0.79		7.1E-04	
19.58	56		-2.72	-0.78			
19.65	82		-1.94	2.88	1.0E-06		
19.96	85	γ-Caprolactone	-2.12	2.40			
20.05	60	Butanoic acid	-3.16	0.21			
20.12	83	3-Octadecene	-1.79	3.54	1.2E-21		
20.28	73		-3.09	-0.52			
20.38	97		-2.80	2.04			
20.91	83	5-Octadecene	-2.37	2.17	1.9E-04		
21.27	69	Farnesol	-2.48	-1.87			
21.33	71		2.42	-1.44			
21.60	82		-0.39	-2.62			
21.83	69		-1.48	-3.04			
22.05	94	Phenol	-3.64	0.03	2.3E-14	1.3E-15	1.7E-12
22.13	58	2-Pentadecanone	-3.49	1.00			
22.16	135	1,2-Benzisothiazol	-2.71	1.29			
22.40	119		-0.29	-1.71			
22.42	107	p-Cresol	-2.95	-1.99			
22.66	82	Tetradecanal	-1.28	-2.54			
22.89	69		-0.87	-3.42			
22.89	93	6-Methyl-2-pyridinecarbaldehyde	-2.13	-0.12			
22.90	69		-0.23	-3.45			
23.83	55		-2.20	1.26			
24.10	117	Indolizine	-2.50	0.10			
