# Supplementary material for: Elevated levels of proinflammatory volatile metabolites in feces of high fat diet fed KK-Ay mice
Source: Sci Rep. 2020 Mar 30;10:5681. doi: 10.1038/s41598-020-62541-7 (PMC7105489; doi:10.1038/s41598-020-62541-7)
Supplement: Supplementary file 6 — Supplementary Table 2 [file 41598_2020_62541_MOESM6_ESM.pdf]

Supplemental Table 2. List of VOCs analyzed by PCA at week 5.

RT (min)	Base peak	Name	PC 1 (20.45%)	PC 2 (15.30%)	*p* (two-way ANOVA) Diet	Lineage	DL
1.42	32		-0.74	-1.51			
1.44	252		-0.21	0.60			
1.48	58	Methylamine, N,N-dimethyl-	-0.67	0.62			
1.56	17		-0.27	0.96			
1.66	29	Acetaldehyde	-0.38	-0.11			
1.84	17		0.14	-0.53			
2.14	41		-2.70	-0.67			
2.15	43	Acetone	-3.40	-1.57			
2.17	43	Propanal, 2-methyl-	1.73	-0.77			
2.58	82		-3.38	1.53			
2.87	43	2-Butanone	-1.28	-1.44			
2.92	31	Methyl Alcohol	-0.94	-0.94			
3.04	41	Butanal, 2-methyl-	-2.23	-0.13			
3.04	43		-2.65	-1.74			
3.05	41		-2.56	-2.43			
3.06	44	Butanal, 3-methyl-	-2.05	-0.20			
3.40	31	Ethanol	0.68	-0.60			
3.46	126		1.14	-2.40			
3.90	43	2-Pentanone	-1.12	-0.01			
4.29	40	Acetonitrile	0.91	0.66			
4.88	75		-1.65	-1.30			
4.99	28		-2.01	1.58			
7.58	18		-0.09	0.30			
8.14	70	Heptanal	-2.94	0.91			
9.20	81	Furan, 2-pentyl-	-2.82	0.18			
9.28	55		-2.29	-2.14		4.7E-02	
10.31	55	1-Pentanol	-3.55	2.23	4.1E-04		
10.51	94	Pyrazine, methyl-	-3.06	-2.09			
10.87	41	Octanal	-2.65	3.19	8.9E-07		
11.13	45	Acetoin	-2.14	0.10			
11.48	43	2-Propanone, 1-hydroxy-	-2.15	-1.19			
12.10	108	Pyrimidine, 4,6-dimethyl-	-2.87	-1.92			
12.54	341		0.39	2.32			
12.90	56	1-Hexanol	-2.37	2.78	2.1E-03		
13.16	126		-2.58	-2.35			
13.65	57	Nonanal	-3.80	1.93	4.7E-04		
15.23	48		0.90	-3.02	3.2E-02		
15.32	57		-0.93	-2.88			
15.94	43	Acetic acid	1.21	-1.96			
16.33	45		1.85	1.83			
16.84	281		-0.15	-0.88			
16.88	106	Benzaldehyde	-2.61	-1.07			
18.11	55		-2.28	0.57			
18.60	74	Propanoic acid	1.34	-0.27			
19.11	43	Propanoic acid, 2-methyl-	-0.50	-0.67			
19.64	67		-1.54	3.57	4.8E-06		
19.64	355		-1.32	-1.95			
19.77	60	Butanoic acid	-1.78	0.25			
20.13	83	8-Heptadecene	-1.94	3.41	8.9E-06		
20.20	74	Butanoic acid, 2-methyl-	-1.85	-2.58			
20.21	60		-0.20	-0.59			

20.81	60		-0.07	-0.42	
22.04	94	Phenol	-3.35	-0.90	
22.13	58	2-Pentadecanone	-3.86	1.16	
22.42	107	p-Cresol	-0.33	-3.31	
22.67	82	Hexadecanal	0.68	-3.61	9.9E-03
24.09	117	Indole	-2.37	-0.45	
