# Supplementary material for: Elevated levels of proinflammatory volatile metabolites in feces of high fat diet fed KK-Ay mice
Source: Sci Rep. 2020 Mar 30;10:5681. doi: 10.1038/s41598-020-62541-7 (PMC7105489; doi:10.1038/s41598-020-62541-7)
Supplement: Supplementary file 7 — Supplementary Table 3 [file 41598_2020_62541_MOESM7_ESM.pdf]

Supplemental Table 3. List of VOCs analyzed by PCA at week 9.

RT (min)	Base peak	Name	PC 1 (30.91%)	PC 2 (16.47%)	*p* (two-way ANOVA) Diet	Lineage	DL
1.41	28		-1.25	1.95			
1.41	28	Acetaldehyde	2.77	-2.00	2.7E-02	7.5E-04	
1.42	44		-0.92	-1.13			
1.44	252		2.11	-1.63		6.2E-03	
1.48	58		-1.84	-1.33			
1.52	17		-2.85	0.86			
1.60	47	Methanethiol	-1.28	-3.43			
1.63	44		-1.14	0.48			
1.65	48		0.16	-2.87			
2.14	43	1-Propen-2ol acetate	0.62	-2.21			
2.14	72		-1.55	-2.26			
2.15	43	Acetone	-3.77	-0.58			
2.56	82	2-pentyl Furan	-1.42	1.72			
2.84	43	2-Butanone	-3.11	-1.51			
2.97	31		-2.64	-1.28			
3.02	41	3-methyl Butanal	-3.58	-0.70			
3.06	44	Pentanal	-3.87	-0.72			
3.43	31		-0.60	-1.71			
3.88	43	2-Pentanone	-3.13	-1.51			
4.26	41		-2.06	0.52			
4.27	41		-3.10	-0.05			
4.91	75		1.48	-2.63			
4.99	28		1.96	-1.77			
7.57	18		0.90	-2.37			
8.12	70	Heptanal	-3.19	1.61			
9.12	80	Pyrazine	-3.28	-0.95		2.5E-02	
9.27	55		-3.44	-0.82		2.9E-02	
10.49	94	methyl Pyrazine,	-3.90	-0.86			
10.84	41	Octanal	-2.93	2.63	1.2E-03		
11.14	45	Acetoin	-2.44	-2.62			
11.48	43	1-hydroxy 2-Propanone	-3.06	-2.03			
11.97	108		-3.33	-1.76			
12.14	108	2,3-dimethyl Pyrazine	-3.72	-1.17			
12.88	56	1-Hexanol	-2.88	2.27	2.2E-02		
13.63	57	Nonanal	-3.23	1.45			
15.20	48		-0.55	-3.16			
16.84	281		1.79	-2.08			
16.84	105		-3.69	-1.10			
18.79	74	Propanoic acid	2.53	-0.57			
19.30	43	2-methyl Propanoic acid	1.13	0.17			
19.64	355		0.11	-0.74			
20.20	74	2-methyl Butanoic acid	-2.84	-0.29			
20.43	43		2.14	-0.98			
20.51	59	Acetamide	-0.78	-2.84			
21.33	71		2.15	-3.21	5.9E-06		
22.03	94	Phenol	-3.14	0.51		3.3E-02	
22.41	107		-1.29	-2.48			
22.67	82		1.38	-2.98			
24.08	117	Indole	-2.83	1.37			
