# Supplementary material for: Elevated levels of proinflammatory volatile metabolites in feces of high fat diet fed KK-Ay mice
Source: Sci Rep. 2020 Mar 30;10:5681. doi: 10.1038/s41598-020-62541-7 (PMC7105489; doi:10.1038/s41598-020-62541-7)
Supplement: Supplementary file 8 — Supplementary Table 4 [file 41598_2020_62541_MOESM8_ESM.pdf]

Supplemental Table 4. List of VOCs analyzed by PCA at week 13.

| RT (min) | Base peak | Name | PC 1 (21.39%) | PC 2 (12.63%) | *p* (two-way ANOVA) | | |
|---|---|---|---|---|---|---|---|
| | | | | | Diet | Lineage | DL |
| 1.43 | 252 | | -0.60 | 2.09 | | | |
| 1.44 | 31 | | 1.62 | -1.90 | | | |
| 1.44 | 43 | 2,3-Butanedione | 0.76 | 3.11 | | | |
| 1.44 | 31 | | -1.65 | 0.27 | | | |
| 1.48 | 58 | Methylamine, N,N-dimethyl- | -0.18 | 1.47 | | | |
| 1.62 | 42 | | 1.72 | -1.16 | | | |
| 1.64 | 44 | Pentanal | 2.28 | 0.83 | | | |
| 1.68 | 29 | Acetaldehyde | 1.32 | 0.86 | | | |
| 2.14 | 43 | Acetone | 3.86 | 1.37 | | | |
| 2.56 | 82 | | 1.55 | -2.44 | | | |
| 2.83 | 43 | 2-Butanone | 2.47 | 0.83 | | | |
| 2.87 | 43 | 2-Hexanone, 4-methyl- | 1.18 | 0.74 | | | |
| 3.00 | 31 | Ethanol | 0.95 | -0.51 | | | |
| 3.03 | 44 | | 2.01 | 1.51 | | | |
| 3.06 | 44 | Butanal, 3-methyl- | 4.05 | 0.71 | | 6.0E-03 | |
| 3.33 | 45 | | 2.00 | 1.61 | | | |
| 3.45 | 31 | 1-Propene, 3-propoxy- | 2.31 | 1.35 | | | |
| 3.87 | 29 | | 2.01 | -2.60 | | | |
| 4.24 | 41 | Acetonitrile | -0.41 | 1.84 | | | |
| 4.93 | 75 | | 0.61 | 3.02 | | | |
| 4.99 | 28 | | -0.39 | -0.91 | | | |
| 7.57 | 18 | | 0.47 | 0.50 | | | |
| 7.80 | 56 | 1-Butanol | -2.01 | -0.07 | | | |
| 8.09 | 70 | Heptanal | 3.22 | -2.34 | | 7.8E-04 | |
| 8.13 | 43 | | -1.78 | 0.78 | | | |
| 9.09 | 80 | 1,3-Diazine | 2.40 | -0.74 | | | |
| 9.21 | 81 | Furan, 2-pentyl- | 1.61 | -2.10 | | | |
| 10.27 | 55 | 1-Pentanol | 2.55 | -0.48 | | | |
| 10.47 | 94 | Pyrazine, methyl- | 3.96 | 0.95 | | 1.4E-02 | |
| 10.81 | 56 | Octanal | 3.28 | -2.35 | 1.6E-16 | 1.6E-16 | 1.6E-16 |
| 10.85 | 57 | | -1.81 | -1.13 | | | |
| 11.07 | 45 | Acetoin | 0.04 | 1.00 | | | |
| 12.11 | 108 | Pyrimidine, 4,6-dimethyl- | 2.99 | 2.63 | | | |
| 12.19 | 107 | | 3.01 | -0.76 | | | |
| 12.86 | 56 | 1-Hexanol | 1.71 | -1.90 | | | |
| 13.08 | 126 | Dimethyl trisulfide | 3.15 | 1.51 | | | |
| 13.62 | 57 | Nonanal | 0.83 | 0.29 | | | |
| 15.16 | 48 | | 1.91 | 2.94 | | | |
| 16.81 | 106 | Benzaldehyde | 3.29 | 1.00 | | | |
| 16.82 | 281 | | -1.36 | 1.49 | | | |
| 19.63 | 355 | | 0.03 | 2.02 | | | |
| 19.74 | 60 | Butanoic acid | -0.08 | -2.63 | | | |
| 20.18 | 60 | | 0.05 | 0.88 | | | |
| 21.32 | 71 | | -1.66 | 2.05 | | | |
| 22.02 | 94 | Phenol | 2.93 | 0.09 | | | |
| 22.40 | 107 | p-Cresol | -2.02 | 1.56 | | | |
| 22.68 | 82 | Hexadecanal | -0.47 | 0.08 | | | |
| 23.25 | 139 | | 2.21 | -0.55 | | | |
| 24.06 | 117 | Indole | 1.56 | -0.59 | | | |
