# Supplementary material for: Elevated levels of proinflammatory volatile metabolites in feces of high fat diet fed KK-Ay mice
Source: Sci Rep. 2020 Mar 30;10:5681. doi: 10.1038/s41598-020-62541-7 (PMC7105489; doi:10.1038/s41598-020-62541-7)
Supplement: Supplementary file 9 — Supplementary Table 5 [file 41598_2020_62541_MOESM9_ESM.pdf]

Supplemental Table 5. List of VOCs analyzed by PCA at week 17.

RT (min)	Base peak	Name	PC 1 (20.33%)	PC 2 (14.09%)	*p* (two-way ANOVA) Diet	Lineage	DL
1.40	32		0.62	-0.08			
1.42	252		1.68	1.35			
1.52	17		2.81	0.29			
1.61	29	Acetaldehyde	1.32	1.56			
1.68	44		2.87	-1.15			
2.12	43	Acetone	4.05	0.06			
2.53	82		1.64	2.12			
2.81	43	2-Butanone	2.97	-1.37			
2.87	31	Methyl Alcohol	-1.63	-0.98			
2.97	57		2.26	-0.12			
3.00	41	2-methyl Butanal	3.53	-0.85			
3.01	44	3-methyl Butanal	2.36	0.60			
3.29	263		-0.58	0.35			
3.34	31	Ethanol	2.66	1.48			
3.83	43	2-Pentanone	-0.48	0.70			
4.18	41	Acetonitrile	0.27	0.86			
4.99	28		-1.24	0.80			
8.04	43		-1.25	1.70			
8.06	43		1.79	2.79			
9.15	81	2-pentyl Furan	0.39	3.71	2.5E-06		
10.77	57	Octanal	0.41	3.45	1.4E-02		
10.96	43		-0.26	-1.74			
13.01	126		1.48	-3.36			
13.54	57	Nonanal	0.83	2.51			
15.07	48		1.20	-3.39			
15.11	43		-1.10	-1.09			
16.72	106	Benzaldehyde	3.37	-1.78			
16.80	281		-3.08	0.77			1.3E-03
17.47	74		-0.76	-1.90			
19.32	60	Butanoic acid	2.52	-1.41			
19.62	355		-2.27	-0.72			
19.71	74	2-methyl Butanoic acid	2.57	0.48			
20.44	60	Heptanoic acid	0.32	-0.31			
22.00	94	Phenol	2.99	1.49	3.2E-02	1.7E-02	1.8E-02
22.39	107	p-Cresol	1.10	-1.03			
24.04	117	Indole	1.38	0.07			
